# Supplementation with carnitine reduces the severity of constipation: a retrospective study of patients with severe motor and intellectual disabilities

**DOI:** 10.3164/jcbn.16-52

**Published:** 2017-03-01

**Authors:** Shinya Murata, Keisuke Inoue, Tomoki Aomatsu, Atsushi Yoden, Hiroshi Tamai

**Affiliations:** 1 Department of Pediatrics, Hirakata City Hospital, 2-14-1 Kinya-honmachi, Hirakata, Osaka 573-1013, Japan; 2 Department of Pediatrics, Osaka Medical College, 2-7 Daigaku-machi, Takatsuki, Osaka 569-8686, Japan

**Keywords:** carnitine, constipation, severe motor and intellectual disabilities

## Abstract

Carnitine is an essential nutrient for the mitochondrial transport of fatty acids. Carnitine deficiency causes a variety of symptoms in multiple organs. Patients with severe motor and intellectual disabilities often have carnitine deficiency. This study aimed to determine the correlation between constipation and carnitine deficiency in them. Patients with severe motor and intellectual disabilities at our hospital were retrospectively reviewed. The correlation between level of free carnitine and severity of constipation was examined. Constipation and non-constipation groups were compared for age; sex; body mass index; bed rest period; use of anti-epileptic drugs, valproate sodium, or enteral nutrition; and serum levels of albumin, pre-albumin, totalcholesterol, free carnitine, folic acid, and trace elements. Moreover, severity of constipation before and after carnitine supplementation was assessed. Twenty-seven patients were enrolled. Of these, 14 were assigned to the constipation group and 13 to the non-constipation group. The free carnitine level was significantly correlated with severity of constipation (R = 0.7604, *p*<0.01). Free carnitine was significantly lower in the constipation compared with the non-constipation group (*p*<0.01). No other significant differences between the groups were found. The severity of constipation was significantly relieved after carnitine supplementation (*p*<0.001). In conclusion, carnitine supplementation could reduce the severity of constipation.

## Introduction

Carnitine, which is acquired mostly through the diet, is an essential nutrient related to the mitochondrial transport of fatty acids. Carnitine deficiency causes a variety of symptoms in multiple organs due to mitochondrial dysfunction.^(1–3)^

Most patients with severe motor and intellectual disabilities have epilepsy and are prescribed anti-epileptic drugs such as valproate sodium, which promotes the excretion of carnitine. Furthermore, as most of these patients cannot feed themselves, they receive enteral nutrition, which has less carnitine content than a normal diet. Use of valproate sodium and most enteral nutrition formulas for an extended time has been reported as a risk factor for carnitine deficiency.^(4–5)^ Therefore, patients with severe motor and intellectual disabilities are known to be at risk of deficiency of carnitine.^(6–7)^

In our clinical practice, we have treated many patients with severe motor and intellectual disabilities who have experienced relief from constipation after supplementation of carnitine. However, to our knowledge, there are few reports of studies designed to examine correlations between gastrointestinal dysmotility and deficiency of carnitine. Therefore, this study aimed to retrospectively review clinical data of patients with severe motor and intellectual disabilities in order to assess our suspicion of a correlation between constipation and carnitine deficiency.

## Methods

### Subjects and Methods

This study was approved by the Ethics Committee of Hirakata City Hospital. Records of patients with severe motor and intellectual disabilities treated at Hirakata City Hospital between January 2012 and December 2014 were retrospectively reviewed. Patients with severe motor and intellectual disabilities were defined as having an inability to move by themselves and IQ<35.^(8)^ Constipation was defined according to the Rome III criteria.^(9)^ Feces forms were classified using the Bristol stool scale.^(10)^ Severity of constipation was classified on a numeric scale defined as, [(# of defecation events per day without use of an enema) + (the numeric Bristol scale score for form of feces)].^(11)^

First, we examined the correlation between level of free carnitine and severity of constipation.

Second, we compared the constipation group with the non-constipation group in terms of age, sex, BMI, bed rest period (years), use of anti-epileptic drugs, use of valproate sodium, use of enteral nutrition, recurrent pulmonary infection (more than twice a year), presence of pressure ulcer, and results of blood tests for nutritional assessment (including serum levels of albumin, pre-albumin, total cholesterol, free carnitine, folic acid, and the trace elements Cu, Fe, Se, and Zn). Blood samples were collected in all cases during times when there were no observed inflammation reactions and no prescribed antibiotics. Carnitine levels also were obtained from patients without motor and intellectual disabilities who had severe chronic constipation requiring disimpaction with an enema on a daily basis.

Third, we compared the frequency of defecation and the form of feces before and after therapeutic supplementation with carnitine.

### Statistical analysis

Between-group comparisons were made using a nonparametric test (Wilcoxon test), and Fisher’s exact test was used for a 2 × 2 contingency table. The correlation between the groups was estimated using Spearman’s rank correlation coefficient test. All statistical analyses were performed using JMP^®^ 12 software (SAS Institute Inc., Cary, NC). Differences having *p* values <0.01 were considered significant.

## Results

A total of 27 patients (19 males, 8 females; age range, 2 to 45 years) with severe motor and intellectual disabilities were enrolled in this study. The constipation group comprised 14 patients, leaving 13 patients in the non-constipation group (Table 1).

In total, 13 patients in the constipation group had epilepsy and were prescribed anti-epileptic drugs (Table 2). Eleven of these (and, thus, 78.6%, 11/14, of the entire constipation group) were taking valproate sodium. In total, 9 (64.3%; 9/14) constipation group patients were administered enteral nutrition formulas with lower carnitine content, such as *RACOL*^®^ (Otsuka Pharmaceutical Co., Ltd., Tokyo, Japan) or *Ensure*^®^ (Abbott Japan Co., Ltd., Tokyo, Japan). In addition, 7 patients in the constipation group had already been taking a medication for constipation, such as magnesium oxide (Table 3).

**Fig. 1:**
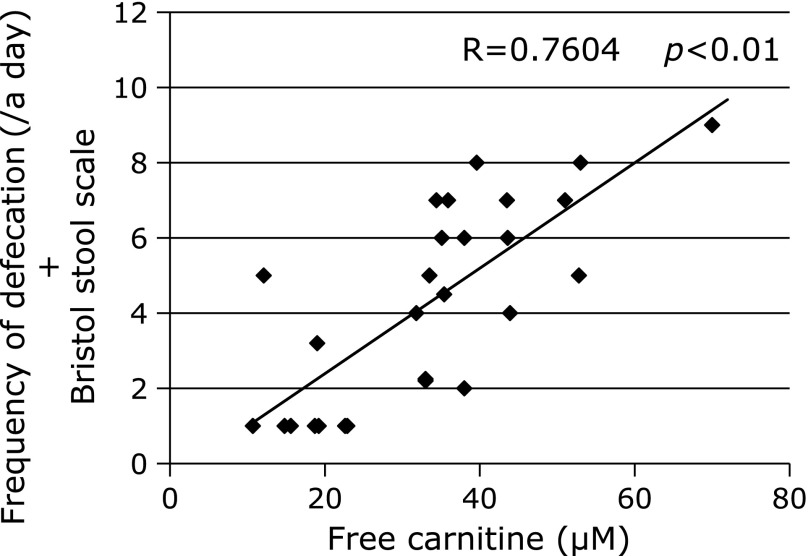
Correlation between free carnitine and constipation severity. Levels of free carnitine were significantly correlated with severity of constipation (R = 0.7604, *p*<0.01).

Levels of free carnitine were significantly correlated with severity of constipation (R = 0.7604, *p*<0.01, Fig. 1).

Levels of free carnitine were significantly lower in the constipation group compared with the non-constipation group (*p*<0.01). There were no significant differences between the two groups for the other data analyzed (Table 3). The patients without motor and intellectual disabilities who had severe constipation were 10 (5 males, 5 females; age range, 2 to 45 years). The free carnitine levels of all these patients were within normal limits (36–74 µM).^(12–13)^

**Fig. 2:**
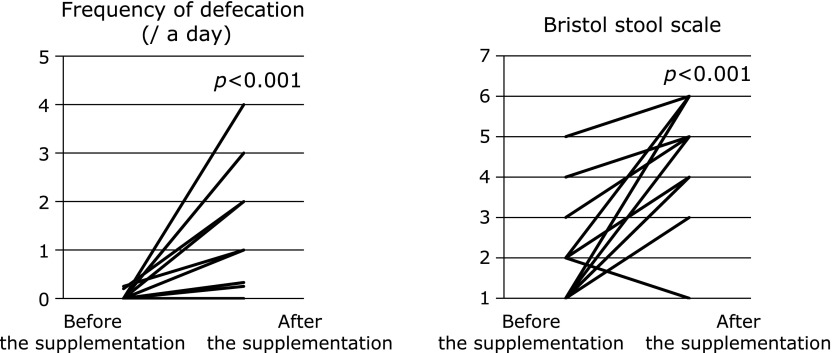
Comparison before and after supplementation of carnitine in the constipation group. The severity of constipation (frequency of defecation and form of feces) was significantly relieved after supplementation with carnitine (*p*<0.001).

The severity of constipation (frequency of defecation and form of feces) in constipation group was significantly relieved after supplementation with 10–50 mg/kg/day carnitine (*p*<0.001, Fig. 2). No other changes of medication were made during the supplementation period.

## Discussion

To our knowledge, this is the first report of a study regarding the correlation between carnitine deficiency and constipation in patients with severe intellectual and motor disability. In patients with severe intellectual and motor disability, the level of free carnitine was significantly correlated with the severity of constipation, and was significantly lower in the constipation group than in the non-constipation group. Moreover, the severity of constipation in the constipation group was significantly relieved after supplementation with carnitine. These results suggest that supplementation with carnitine could reduce the severity of constipation and that carnitine deficiency could be one of the causes of constipation.

Furthermore, some patients in the constipation group resisted taking an ordinary dose of laxatives, but their severe constipation was relieved after supplementation with carnitine. These findings suggest the possibility that carnitine deficiency is fairly common among those who cannot ingest a diet containing sufficient carnitine, have severe constipation, and resist laxative use, such as elderly, bedridden people. Further studies are necessary to assess this possibility.

Carnitine is an essential factor involved in the mitochondrial transport of fatty acids, and carnitine deficiency causes mitochondrial dysfunction. Signs and symptoms of carnitine deficiency occur in multiple organs, and include disturbance of consciousness, convulsions, muscle weakness, cardiomyopathy, fatty liver, and hypoglycemia.^(1–3)^ Therefore, carnitine deficiency appears to influence gastrointestinal motility. Weaver *et al.*^(14)^ reported that an infant with delayed development and peripheral myopathy, nourished on a soy-based liquid diet deficient in carnitine, had gastrointestinal dysmotility manifested by postprandial vomiting, oral drooling, delayed gastric emptying and infrequent bowel movements. After dietary supplementation with carnitine the gastrointestinal symptoms resolved and serum carnitine increased to within normal limits. Mostafa *et al.*^(15)^ reported that autistic patients with gastrointestinal manifestations had significantly decreased levels of serum carnitine compared with patients without such manifestations. In addition, autistic patients with gastrointestinal manifestations had significantly lower serum carnitine compared with patients without such manifestations. Most of the gastrointestinal manifestations was constipation (87.5%, 42/48). Those reports are consistent with the correlation between carnitine deficiency and constipation seen in the current study.

However, in the patients without motor and intellectual disabilities who had severe constipation, free carnitine levels were within normal limits. We thought that these patients could eat a diet with a sufficient content of carnitine, in foods such as beef and chicken. Constipation is not consisted of single factor. Constipation is a complex syndrome often involving multiple causative factors. In this study, we could not sufficiently examine the multiple causative factors, such as underlying disease, drugs, nutrient intake, or degree of motor paralysis. Therefore, we could not completely rule out the possibility that carnitine had a simple laxative effect. This is an important limitation of our study. To assess this issue, further studies of a larger population are needed.

In conclusion, supplementation with carnitine could reduce severity of constipation in patients with severe intellectual and motor disability and carnitine deficiency could be one of the causes of constipation. There is possibility that carnitine deficiency is latent also in those who cannot do sufficient oral intake. For such patients with severe constipation resisted against laxative, it may be necessary to add carnitine deficiency to the differential diagnosis.

## Figures and Tables

**Table 1:** Patient characteristics

**Background**	
Age (years)	21 (7–25)
Sex (male/female)	19/8
BMI	14 (12.4–17.6)
Bed rest period	16 (6–25)
Anti-epileptic drug (+/–)	24/3
Valproate sodium (+/–)	16/11
Main route of administration of nutrition (Enteral nutrition/Oral)	16/11
Recurrence pulmonary infection (+/–)	7/20
Pressure ulcer (+/–)	9/18
**Blood sample test for nutritional assessment**	
Albumin (g/dl)	4 (3.6–4.2)
Prealbumin (mg/dl)	20 (18.3–23)
T-chol (mg/dl)	152 (130–171)
Cu (µg/dl)	129 (120–135)
Fe (µg/dl)	63 (47–75)
Se (µg/L)	76 (58–88)
Zn (µg/dl)	61 (50–68)
Folic acid (ng/ml)	12.4 (6.9–18.2)
Free carnitine (µmol/L)	34.4 (19.2–43.5)

**Table 2:** Comparison between constipation group and non-constipation group

	Constipation group (*n* = 14)	Non-constipation group (*n* = 13)	*p*
**Background**			
Age (years)	17.5 (6.8–25.8)	21 (9.5–26.5)	0.7154
Sex (male/female)	9/5	10/3	0.6776
BMI	13.8 (11.8–15.3)	17.1 (13.8–18.1)	0.0941
Bed rest period	11.5 (6–24.3)	20 (8–25)	0.4502
Anti-epileptic drug (+/–)	13/1	11/2	0.5956
Valproate sodium (+/–)	11/3	5/8	0.0542
Main route of administration of nutrition (Enteral nutrition/Oral)	9/5	7/6	0.7036
Recurrence pulmonary infection (+/–)	6/8	1/12	0.0768
Pressure ulcer (+/–)	7/7	2/11	0.1032
**Blood sample test for nutritional assessment**			
Albumin (g/dl)	3.9 (3.3–4.3)	4 (3.7–4.2)	0.8646
Prealbumin (mg/dl)	20 (18.2–21.6)	22 (18.1–25.2)	0.3192
T-chol (mg/dl)	158.5 (123–164.3)	145 (130.5–183.5)	0.6274
Cu (µg/dl)	130.5 (115.3–135.8)	128 (120.5–135.5)	0.9032
Fe (µg/dl)	61.5 (46–79.5)	65 (46–73.5)	0.9226
Se (µg/L)	69 (36–102.5)	73 (71–87.5)	0.6793
Zn (µg/dl)	56 (43–69.3)	61 (53.5–68)	0.3428
Folic acid (ng/ml)	10.5 (5.9–18.3)	12.4 (9.2–19.2)	0.3957
Free carnitine (µmol/L)	20.9 (15.4–33)	43.5 (35.7–51.9)	<0.01

**Table 3:** Characteristics of the constipation group

Case	Age (years), Sex	Basal disease	Bed rest period (years)	Antiepileptic drugs	Main route of administration of nutrition	Use of laxatives before carnitine supplementation
1	5, male	West syndrome	5	VPA, CBZ, CLB, TPN	Enteral nutrition -NG tube (*RACOL*^®^)	enema
2	6, male	Periventricular leukomalacia	6	VPA	Oral intake	magnesium oxide, enema
3	6, female	Periventricular leukomalacia	6	VPA, CBZ, CZP	Enteral nutrition -PEG (*RACOL*^®^)	magnesium oxide
4	7, female	Acute encephalopathy	6	VPA, CZP, TPN	Enteral nutrition -PEG (*RACOL*^®^)	magnesium oxide
5	8, male	Tuberous sclerosis	8	VPA, CLB, LTG	Oral intake	enema
6	11, male	Acute encephalopathy	10	PB, ZNS	Enteral nutrition -PEG (*Ensure*^®^)	enema
7	14, male	Pelizaeus-Merzbacher disease	13	VPA	Oral intake	enema
8	21, female	Juvenile neuronal ceroid lipofuscinosis	19	VPA, CZP, ZNS	Enteral nutrition -PEG (*RACOL*^®^)	magnesium oxide, enema
9	22, male	Bacterial meningitis	21	NZP	Enteral nutrition -NG tube (*Ensure*^®^)	enema
10	23, male	Cerebral infarction	4	VPA, LEV	Enteral nutrition -PEG (*RACOL*^®^)	(–)
11	25, male	Bacterial meningitis	24	VPA, CBZ, LEV	Enteral nutrition -NG tube (*Ensure*^®^)	magnesium oxide, enema
12	28, female	Periventricular leukomalacia	28	(–)	Enteral nutrition -PEG (*RACOL*^®^)	enema
13	36, male	Cerebral palsy	36	VPA, NZP, ZNS	Oral intake	magnesium oxide, enema
14	45, female	Cerebral palsy	45	VPA, CBZ, LEV	Oral intake	magnesium oxide, enema

CBZ, carbamazepine; CLB, clobazam; CZP, clonazepam; LEV, levetiracetam; LTG, lamotrigine; NZP, nitrazepam; PB, phenobarbital; VPA, valproate sodium; ZNS, zonisamide.
